# Complete chloroplast genome sequences of two species of *Chloris* grass, *Chloris truncata* Sw. and *Chloris virgata* R.Br

**DOI:** 10.1080/23802359.2016.1266705

**Published:** 2017-01-04

**Authors:** James P. Hereward, Jeff A. Werth, David F. Thornby, Michelle Keenan, Bhagirath Singh Chauhan, Gimme H. Walter

**Affiliations:** a School of Biological Sciences, The University of Queensland, Brisbane, Australia;; b Queensland Department of Agriculture and Fisheries, Leslie Research Centre, Toowoomba, Australia;; c Innokas Intellectual Services, Coomera, Australia;; d The Centre for Plant Science, Queensland Alliance for Agriculture and Food Innovation (QAAFI), The University of Queensland, Gatton, Australia

**Keywords:** *Chloris*, glyphosate resistance, Australia, weed

## Abstract

*Chloris truncata* (windmill grass) and *Chloris virgata* (feathertop Rhodes grass) are both weedy grass species that have developed resistance to glyphosate in Australia. This paper describes the complete chloroplast genomes of these two species generated by high throughput shotgun sequencing. The chloroplast genome of *C. truncata* is 135,584 bp and *C. virgata* is 134,561 bp; both have a GC content of 38%. The gene content and order followed the conserved pattern observed across the subfamily Chloridoideae.

*Chloris truncata* Sw. (windmill grass) is considered native to Australia (Michael et al. 2012), whereas *Chloris virgata* R. Br. (feathertop Rhodes grass) is considered naturalised (van Klinken et al. 2004). Both species were identified as having a high risk of evolving glyphosate resistance due to high seed production and other life history characteristics (Werth et al. 2011). *Chloris truncata* was confirmed as glyphosate resistant in 2010 and *C. virgata* was confirmed resistant in 2015 in Australia (Heap 2016; Preston 2016). High-throughput shotgun sequencing was conducted on genomic DNA from these two species to develop molecular markers for population genetics analysis. This sequencing also yielded the complete chloroplast sequences for these two species which are reported here.

*Chloris truncata* seeds were collected near Trangie in NSW, Australia, and *C. virgata* seeds were collected near Aberdeen Road, Wyaga, Queensland, Australia. Both populations were subsequently confirmed as glyphosate-resistant and GPS co-ordinates have been withheld to protect landholders. Plants were grown from these seeds and DNA was extracted from leaf material, seed-lines are maintained at QDAF, Toowoomba, representative vouchers and DNA samples of each species are held at the University of Queensland (*C. truncata =* WR01, *C. virgata* = VA2). Genomic sequencing libraries were constructed using the NebNext Ultra DNA kit (New England Biolabs, Ipswich, MA) and PE125 Illumina sequencing was performed by Novogene (Beijing, China).

The chloroplast sequences were assembled in Geneious v9.1.3 (http://www.geneious.com, Kearse et al. 2012) by first mapping reads to the complete *Chloris barbata* Sw. chloroplast sequence (genbank accession KT168393, Duvall et al. 2016), followed by *de-novo* assembly of chloroplast reads. Annotations were made based on the *C. barbata* reference and then checked manually. All available complete chloroplast sequences from the subfamily Chloridoideae were downloaded from genbank and aligned with the new *Chloris* chloroplasts. *Centropodia glauca* (Nees) T.A. Cope (Accession KT168383) was included as an outgroup. The most appropriate substitution model was found to be GTR + I + G using jmodeltest2 (Darriba et al. 2012). A Bayesian phylogenetic tree (Figure 1) was produced using this model in MrBayes (Huelsenbrook & Ronquist 2001).

**Figure 1.**
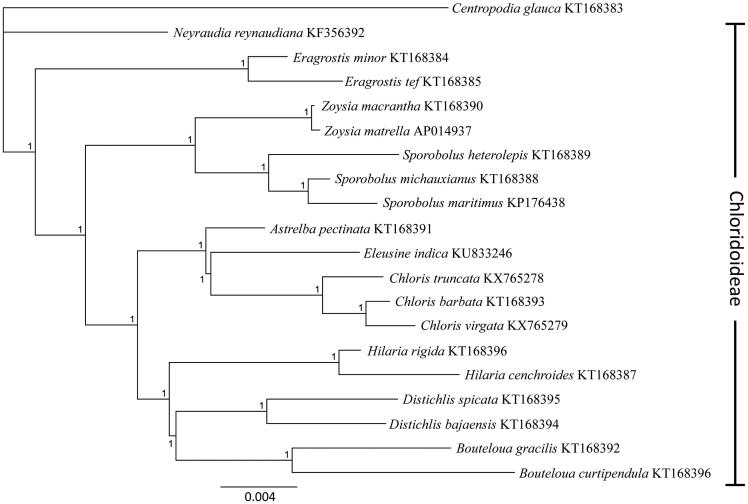
Phylogenetic tree produced using Bayesian estimation (Mr. Bayes) on all complete chloroplast genomes available from subfamily Chloridoideae under the GTR + I + G model, node labels indicate the posterior probability after 1×106 iterations.

The two new *Chloris* chloroplasts have the same gene content, order and inverted repeat size as *C. barbata*, and overall there were <2.2% sequence differences between these three species. The *C. truncata* plastid was larger (135,584 bp) than *C. barbata* (135,372 bp), but *C. virgata* plastome was smaller (134,561 bp). With the exception of the RpoC2 gene, which is known to be of variable length in grasses (Cummings et al. 1994), all insertions and deletions were in the intergenic regions. The phylogenetic tree recovered the same overall topology as previous whole-chloroplast analysis (Duvall et al. 2016) and multi-gene phylogenies (Peterson et al. 2010).

The fully annotated sequences have been deposited at genbank (*C. virgata* = KX765279, *C. truncata* = KX765278). These resources will allow the filtering of chloroplast sequence from future genomics work on these two species and enable further phylogenomic analysis of the genus *Chloris* following the completion of additional plastid genomes.
